# Bulk metallic glass composite with good tensile ductility, high strength and large elastic strain limit

**DOI:** 10.1038/srep05302

**Published:** 2014-06-16

**Authors:** Fu-Fa Wu, K. C. Chan, Song-Shan Jiang, Shun-Hua Chen, Gang Wang

**Affiliations:** 1 Advanced Manufacturing Technology Research Centre, Department of Industrial and Systems Engineering, The Hong Kong Polytechnic University, Kowloon, Hong Kong, China; 2 School of Materials Science and Engineering, Liaoning University of Technology, Jinzhou 121001, China; 3 Laboratory for Microstructures, Shanghai University, Shanghai 200444, China

## Abstract

Bulk metallic glasses exhibit high strength and large elastic strain limit but have no tensile ductility. However, bulk metallic glass composites reinforced by in-situ dendrites possess significantly improved toughness but at the expense of high strength and large elastic strain limit. Here, we report a bulk metallic glass composite with strong strain-hardening capability and large elastic strain limit. It was found that, by plastic predeformation, the bulk metallic glass composite can exhibit both a large elastic strain limit and high strength under tension. These unique elastic mechanical properties are attributed to the reversible B2↔B19′ phase transformation and the plastic-predeformation-induced complicated stress state in the metallic glass matrix and the second phase. These findings are significant for the design and application of bulk metallic glass composites with excellent mechanical properties.

The elastic strain limit, along with the elastic limit (the highest stress at which permanent deformation will not occur), is an important factor for engineering materials^1^. By eliminating the extrinsic flaws and decreasing the internal structural defects, submicrosized metallic glasses (MGs) can reach an outstandingly large elastic strain limit of more than 3%^2^,^3^,^4^,^5^. The elastic strain limit for bulk metallic glasses (BMGs) is almost 2%, which is also significantly high in contrast with common engineering materials, though smaller than nanosized MGs. However, BMGs usually fail catastrophically by the fast propagation of a major shear band, leaving zero global plastic strain under tension^6^,^7^,^8^. Therefore, second phases are in-situ or ex-situ introduced to reinforce the MG matrices^9^,^10^,^11^,^12^,^13^, such as dendrite-reinforced Zr- or Ti-based bulk metallic glass composites (BMGCs)^14^,^15^,^16^,^17^,^18^.

Though their toughness or ductility is significantly increased, the yield strength and the elastic strain limit of BMGCs are decreased dramatically in contrast to monolithic BMGs^15^,^18^,^19^. Usually, the second phases have a relatively small elastic strain limit (not larger than 1%), which results in the premature yield of the BMGCs. Furthermore, the volume fraction of the soft second phase should be higher than 50% to toughen the MG matrix, which severely decreases the elastic strain limit and the strength of BMGCs^14^,^15^,^16^,^17^,^18^. Therefore, a suitable second phase is the key factor for improving the strength, elastic strain limit and ductility of BMGCs. To conserve the large strain limit of BMGs, the second phases also should have a large strain limit not less than 2%. Further, to keep the high strength of BMGs, the second phases should originally have a high enough strength or the soft second phases can be strengthened to a higher strength. It is noted that, in NiTi shape memory alloys, the metastable B2 phase can be strain hardened from less than 100 MPa to more than 1200 MPa, and can undergo a reversible phase transformation of bcc B2↔monoclinic B19’, which endows the alloy with high yield strength and superelasticity^20^. Therefore, by adding a metastable B2 phase and suitable plastic predeformation, the B2 reinforced BMGCs^21^,^22^,^23^,^24^,^25^,^26^,^27^,^28^,^29^ should exhibit a good match in elasticity, strength, and ductility. For example, CuZr-based BMGC with nanosized B2 phase exhibited tensile ductility^30^, and NiTi-based BMGC also showed a good combination of high strength and large pseudo-elasticity under compression^31^,^32^.

In this work, we report a metastable B2 reinforced BMGC (B2-BMGC) with excellent plastic deformation capability under tension. We demonstrate that the B2 phase effectively improves the plastic deformation capability of the B2-BMGC under tension, and the plastic predeformation endows this B2-BMGC with high strength and a large elastic strain limit by reversible B2↔B19’ phase transformation.

## Results

### Microstructure of as-cast B2-BMGC

**Figure 1:**
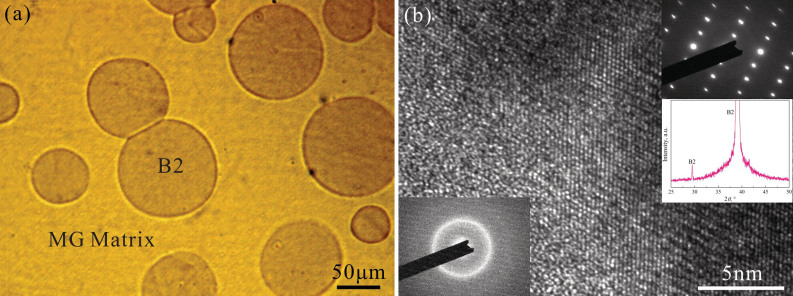
(a) OM metallograph showing the microstructure of the as-cast B2-BMGC. The round particles are the B2 phase, and the matrix is amorphous MG. (b) HRTEM image of the interface between the B2 phase and the amorphous matrix. Inset at the lower left corner shows the selected area diffraction pattern of the amorphous structure; the inset at the upper right corner shows the selected area diffraction pattern of the crystalline structure, and the inset at the middle right shows the X-ray diffraction pattern of the B2-BMGC.

Figure 1a shows an optical metallograph of the microstructure of the as-cast B2-BMGC. The round and dark particles are B2 crystals, which are homogeneously distributed in the amorphous MG matrix. The average chemical compositions for the B2 phase and the amorphous MG matrix are detected by energy-dispersive spectroscopy (EDS) to be Zr_52.1_Cu_41.7_Al_3.9_Nb_2.3_ and Zr_50.7_Cu_42.2_Al_4.1_Nb_3.0_, respectively. It is clear that the difference between the chemical compositions of the B2 phase and the amorphous MG matrix is very small, which indicates the precipitation of B2 phase from the melt during solidification does not involve strong element diffusion, like that usually occurring in in-situ dendrite-reinforced BMGCs^14^. The volume fraction of the B2 phase is about 32.2% and the average grain size of the B2 particles is 67 ± 5 μm in diameter. Figure 1b shows the high-resolution transmission electron microscope (HRTEM) image of the interface between the B2 phase and the amorphous MG matrix. The electron diffraction patterns show that the disorder region is fully amorphous (see lower left inset in Fig. 1b) and the adjacent region is of long-range order (see upper right inset in Fig. 1b). The crystalline phase is further confirmed to be B2 phase with a body-centered cubic structure by the use of X-ray diffraction (see middle right inset in Fig. 1b).

### Tensile deformation of as-cast B2-BMGC

**Figure 2:**
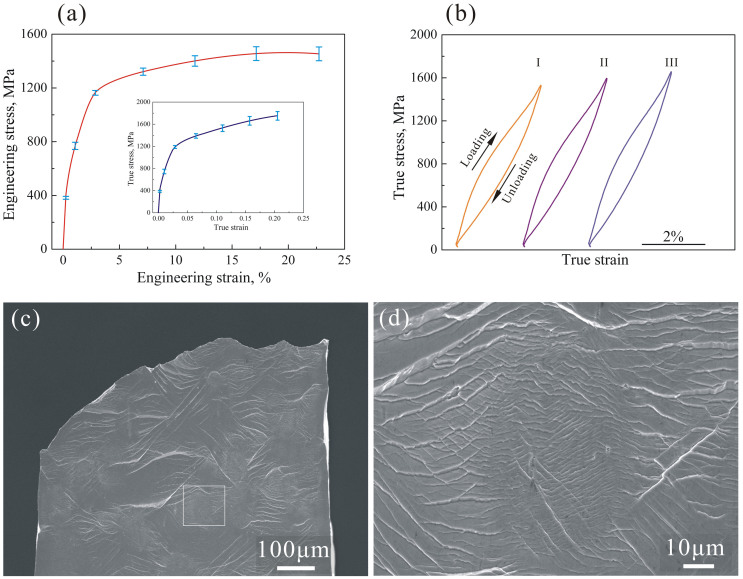
(a) Engineering tensile stress-strain curve of the as-cast B2-BMGC showing large homogeneous plastic deformation capability. The inset in (a) is the true stress-strain curve of the as-cast B2-BMGC showing significant strain-hardening. The error bars were based on standard deviation. (b) cyclic loading stress-strain curves showing large elastic strain limit and nonlinear elasticity for the (I) 10.2%, (II) 12.6%, and (III) 15.0% plastically predeformed B2-BMGCs. (c) and (d) Deformation feature of the as-cast B2-BMGC after tensile fracture.

Figure 2a shows the engineering stress-strain curve of the as-cast B2-reinforced BMGC subjected to tensile loading. It is seen that the sample underwent a large homogeneous plastic deformation with a total engineering strain of 22.3% on average (the maximum engineering plastic strain before fracture was 19.3%). Close examination indicates that the stress-strain curve can be divided into four stages. Firstly, the sample undergoes an initial linear elastic stage under relatively low stress. In this region, both the B2 phase and the MG matrix synchronously deform elastically. Secondly, the sample slightly yields at 387 MPa with recoverable elastic strain of about 0.2%. In this region, the MG matrix is still in elastic state, but the B2 phase reaches its yield point and begins to plastically deform. Thirdly, the sample yields apparently at a stress of 1100 MPa and is strain-hardened to more than 1400 MPa at an engineering strain of 17.8%. Fourthly, a small stress decrease starts and is a prelude to the beginning of tensile instability and final fracture. The inset in Fig. 2a is the true stress-strain curve corresponding to the engineering curve for the as-cast B2-BMGC. It demonstrates that the as-cast B2-BMGC possesses a strong strain-hardening capability under tension: beginning from the apparent yield stress of *σ_S_* = 1100 MPa, the true stress continues increasing to the fracture strength of 1765 MPa, as seen in the inset of Fig. 2a. The average strain-hardening rate (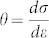) in the smooth region (true strain between 0.05 and 0.15) is 3366 MPa, and the normalized strain-hardening rate (*θ_0_* = *θ*/*σ_S_*) is 3.1, which is higher than most previously reported BMGCs^28^.

### Elastic response of plastically predeformed B2-BMGC

Figure 2b shows the true tensile stress-strain curves of the B2-BMGC after plastic predeformation with a total engineering tensile strain of 10.2%, 12.6%, and 15.0%, as marked with cycles I, II, and III, respectively. It indicates that the B2-BMGC exhibits nonlinear elastic stress-strain behavior, which is significantly different from the linear elasticity of typical monolithic BMGs and other BMGCs^6^. The elastic strain limit is about 2.7%, which is remarkably larger than that (about 2%) of monolithic BMGs or that (usually small than 2%) of other reported BMGCs, and far larger than that (0.2%) of the as-cast B2-BMGC. Further examination reveals that the nonlinear elastic stress-strain curves can be divided into three segments: an initial linear segment, a following parabolic segment, and a final steep segment. The first linear segment is attributed to the synchronized linear elastic response of both the MG matrix and the B2 phase at relatively low stress. The second parabolic segment demonstrates obvious nonlinear elastic stress-strain behavior and a continuously reducing slope, which is mainly triggered by the B2→B19’ phase transformation at relatively high stress level. The third steep segment reflects that the B2→B19’ phase transformation has reached the saturation. During this segment, the transformed B19’, the residual B2, and the MG matrix all elastically deform synchronously.

### Structural evolution of B2-BMGC during plastic predeformation and elastically reloading

**Figure 3:**
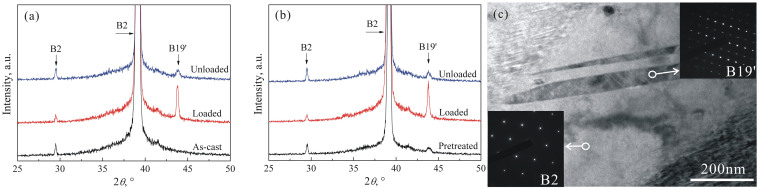
XRD patterns showing the phase transformation during (a) plastic predeformation and (b) elastic reloading. During plastic predeformation, a sharp transformation of B2→B19’ is detected, and a residual B19’ is also detected after plastic predeformation, as shown in (a). During the elastic reloading of the plastically predeformed B2-BMGC, the B19’ peak is strengthened, indicating that more B2 is transformed to B19’, and a reversible transformation of B19’→B2 is observed, as shown in (b). (c) TEM image showing the B19' phase transformed from B2 phase in the plastically predeformed B2-BMGC.

Figure 3a shows the X-ray diffraction (XRD) patterns of the B2-BMGC during plastic predeformation. For the as-cast B2-BMGC, it shows that a strong diffraction peak (2*θ* = 39.1°) of the B2 phase superimposed in the scattering diffraction peak of the amorphous MG matrix, as seen in Fig. 3a. When plastically predeformed with a total engineering tensile strain of 10.2%, a sharp diffraction peak appears at 2*θ* = 43.8°, which is confirmed to be the B2→B19’ phase transformation. After removing the load, the diffraction peak at 2*θ* = 43.8° decreased in intensity, but still existed, implying that the B19’ remained although some B19’ transformed backwards to B2, as shown in Fig. 3a and 3c. Figure 3b shows the XRD patterns of the plastically predeformed B2-BMGC during elastically reloading. It shows that, with the load increasing, the diffraction peak at 2*θ* = 43.8° was strengthened, which means more of the B2 phase was transformed to the B19’ phase. Once the load decreased, the diffraction peak of the B19’ phase weakened again, as in its original profile (Fig. 3b). This structural evolution revealed by XRD is consistent with the nonlinear elastic stress-strain behavior of the plastically predeformed B2-BMGC (see Fig. 2b).

## Discussion

### Large plastic stability of as-cast B2-BMGC under tension

The above results indicate that the as-cast B2-BMGC can undergo large tensile plastic deformation. The plastic strain before fracture is about 19.3%, and the normalized strain-hardenging rate (*θ_0_* = *θ*/*σ_S_*) is about 3.1. The prominent tensile plastic deformation capability of the as-cast B2-BMGC can be attributed to the high strain-hardening capability of the B2 phase and its effectiveness in activating multiple shear bands in the MG matrix. It was previously reported that the normalized strain-hardening rate of CuZr-based B2 phase was about 17.4, which is far larger than that of β dendrite in-situ formed in Ti- or Zr-based BMGCs^28^. For instance, the normalized strain-hardening rate of the β dendrite Zr_71_Ti_16.3_Nb_10_Cu_1.8_Ni_0.9_ is 1.7, which is only one tenth of the present B2 phase^28^. From the viewpoint of the microstructure, the B2→B19 phase transformation can produce hierachical deformation structures with macrotwin, microtwin, stacking fault and dislocation^33^,^34^, which yields dense stress-concentration sites at the interface and can trigger profuse tiny multiple shear bands in the MG matrix^24^,^29^,^35^. Even shear bands excited from one B2 crystal can have different propagation directions and can intersected with each other, as shown in Fig. 2d. Furthermore, these shear bands will propagate forwards and intersect with those excited from the neighbouring B2/MG interfaces. Therefore, the B2 phase is a very effective exciter for the initiation of multiple shear bands in the MG matrix. However, small β dendrites can only excite a few shear bands and can easily cut off by the propagating shear bands. The shear bands around one dendrite almost have the same propagation direction, and their interaction among them is very limited^28^,^36^,^37^.

### Large elastic strain limit of plastically predeformed B2-BMGC

**Figure 4:**
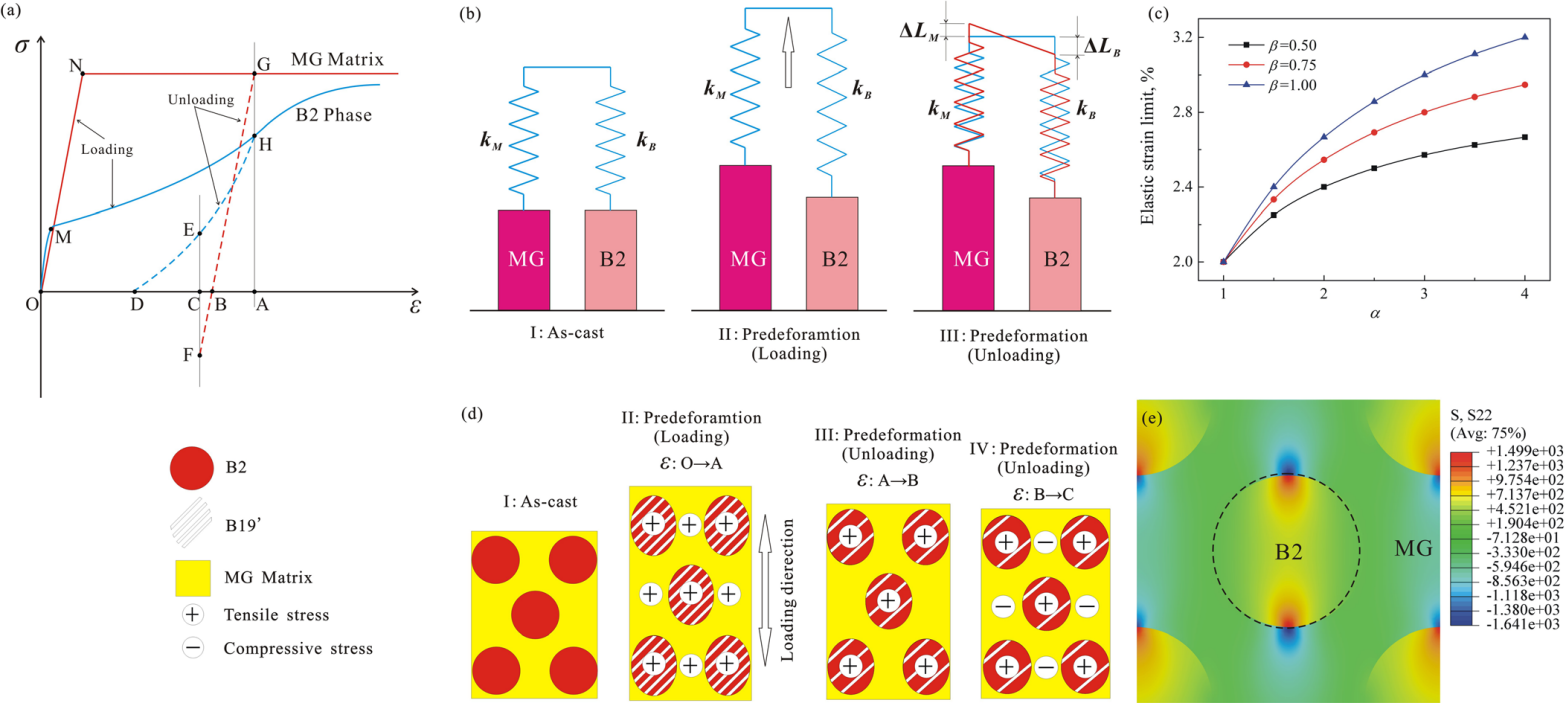
(a) Schematic illustration of the loading history for the B2-BMGC during plastic predeformation and elastic reloading. (b) Model of parallel connection of two rigid-plastic bodies series-connected with ideal-elastic bodies for the B2-BMGC during plastically predeformation. (c) The elastic strain limit of the B2-BMGC related to *α* and *β*. (d) Schematic illustration of stress-strain status of B2 phase and MG matrix in the B2-BMGC during plastic predeformation. (e) FEM simulation result showing the stress state of the plastically predeformed B2-BMGC.

Usually, the elastic strain limit of monolithic BMGs is about 2.0%^38^,^39^, while the elastic strain limit of classical BMGCs is much smaller than 2.0%^17^. However, the present plastically predeformed B2-BMGC has a large elastic strain limit of 2.7%. This unique deformation behavior of the B2-BMGC can be explained as follows. Figure 4a schematically shows the loading history of the B2-BMGC during plastically predeformation: lines ON, NG, and GB are the stress-strain curves for the elastic deformation, plastic deformation, and elastic recovery of the MG matrix; lines OM, MH, HD are the stress-strain curves for the elastic deformation, plastic deformation, and elastic recovery of the B2 phase; point C is the final stress balance point after unloading. Both the MG matrix and the B2 phase can be regarded as a parallel connection of two rigid-plastic bodies series-connected with ideal-elastic bodies, as shown in Fig. 4b–I. For the as-cast B2-BMGC, it is assumed that the MG matrix and the B2 phase have the same length. When plastically predeformed to a certain strain, due to the B2↔B19’ reverse phase transformation, the B2 phase has a larger elastic strain limit than the MG matrix, while the MG matrix has a larger plastic strain than the B2 phase, though they have the same total strain, i.e. 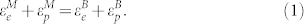
Here 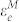 is the elastic strain limit of the MG matrix, 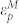 the plastic strain of the MG matrix, 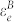 the elastic strain limit of the B2 phase, and 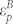 the plastic strain of the B2 phase, as shown in Fig. 4b-II. After plastic predeformation, the external force is removed, and the elastic strain will tend to recover. In an ideal free-standing situation, both MG matrix and B2 phase will recover to the zero stress state, as shown in the red spring in Fig. 4b-III. However, due to the elastic strain mismatch and the mutual constraint, the recovery of the B2 phase will be inhibited by the MG matrix, while the recovery of the MG matrix will be promoted by the recovery of the B2 phase. Therefore, the MG matrix will be in a compressive stress state and the B2 phase will be in a tensile stress state, as shown in the blue spring in Fig. 4b-III. Thus, we have 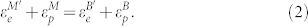
Here 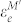 and 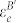 are the residual elastic strains in the MG matrix and the B2 phase, respectively. According to the static balance between the MG matrix and the B2 phase, one gets 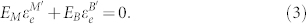
*E_M_* and *E_B_* are the elastic modulus of the MG matrix and the B2 phase after plastic predeformation. Substituting Equations (1) and (2) to Equation (3), we get the residual elastic strain in the MG matrix as 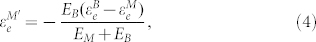
and the residual elastic strain in the B2 phase is 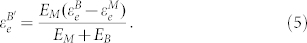
Obviously, Equations (4) and (5) demonstrate that the residual elastic strain in the MG matrix is compressive, while the residual elastic strain in the B2 phase is tensile. Due to the elastic strain limit of the MG matrix being smaller than that of B2 phase, the elastic strain limit of the B2-BMGC will be decided by the MG matrix. Therefore, when subject to tensile loading, the apparent elastic strain limit of the plastically predeformed B2-BMGC is 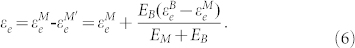
Subsituting 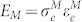
and 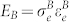 to Equation (6), we get 
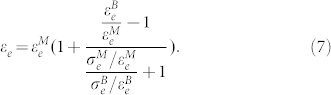
Given 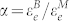 and 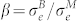, Equation (7) can be simplified as 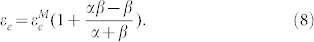
Here we define *α* as the ratio of elastic strain limit between the B2 phase and the MG matrix that reflects the relative elastic recovery capability of the B2 phase, and *β* represents the ratio of strength between the B2 phase and the MG matrix, reflecting the strengthening effect. According to Equation (8), we can get the elastic strain limit of plastically predeformed B2-BMGC related to the factors *α* and *β*, as shown in Fig. 4c. For a given *β*, the elastic strain limit of the plastically predeformed B2-BMGC will monotonically increase with *α*, which implies that increasing the elastic recovery capability of the B2 phase will increase the elastic strain limit of the B2-BMGC. For example, when *β* = 0.50, the elastic strain limit of the plastically predeformed B2-BMGC can increase from 2.0% to 2.7%, with *α* increasing from 1 to 4. When *β* = 0.75, the elastic strain limit of the plastically predeformed B2-BMGC can increase from 2.0% to 2.9%, with *α* ranging from 1 to 4. When *β* = 1.00, the elastic strain limit of the plastically predeformed B2-BMGC can increase from 2.0% to 3.2%. Obviously, large values of *α* and *β* can remarkably increase the elastic strain limit of the B2-BMGC. Therefore, Eq. 8 and Fig. 4c demonstrates that the elastic strain limit of a plastically predeformed B2-BMGC can be well tailored by tuning *α* and *β*.

With a predeformation of more than 10% total strain (elastic and plastic deformation), the B2 phase underwent a large plastic deformation and was strain-hardened to high strength, as schematically shown in Fig. 4a. Meanwhile, the MG matrix was also plastically deformed and its strength invariable^40^. When unloading, the B19’ phase gradually reversely transformed to the B2 phase (see XRD patterns in Fig. 3a). Due to B19’→B2 reverse transformation, the B2 phase recovered to a large quasi-elastic strain *ε_AD_* of more than 6%^41^, which is much larger than the 2% of the MG. Clearly, in the plastically predeformed B2-BMGC, there is an elastic strain recovery mismatch, so both could not elastically recover freely. At the beginning of elastic recovery, both the B2 phase and the MG matrix could elastically recover to *ε_B_*, where the MG matrix elastically recovered to a zero stress status while the B2 phase was still at a tensile stress of *σ_E_*. Then, the B2 phase further recovered forwards the zero stress but the MG matrix would inhibit this elastic recovery, leaving the MG matrix to be squeezed into a compressive stress state. Therefore, the plastically predeformed B2-BMGC stayed in a complicated microsopical internal stress state: the B2 phase stayed in a tensile stress (*σ_E_*) with a corresponding elastic tensile strain (*ε_DC_*), and the MG matrix stayed in a compressive stress (*σ_F_*) with a corresponding elastic compressive strain (*ε_BC_*), as shown in Fig. 4a, 4b, and 4d-III. The finite element modeling (FEM) result in Fig. 4e shows that the stress state in the plastically predeformed B2-BMGC is truly compressive in the MG matrix but tensile in the B2 phase, which is basically consistent with the above analysis in Fig. 4a, 4b, and 4d, as well as the stress concentration near the interface.

When reloaded, the compressed MG matrix firstly elastically recovered to the zero stress state from the originally compressive stress state. Further loading caused the MG matrix to be in a tensile stress state. Meanwhile, the former B2 phase recovering from B19’ in the plastically predeformed B2-BMGC would transform to B19’ again. Theoretically, the apparent elastic strain limit of the plastically predeformed B2-BMGC is |*ε_BC_*|+*ε_BA_ = * |0~−2%|+2% = 0~4% (ε_BC_ = 2% is an ideal value that can not be reached unless the B2 phase possesses a high enough strength and superelasticity), as shown in Fig. 4a. Obviously, a large elastic strain limit of about 2.7% for the plastically predeformed B2-BMGC is reasonable.

**Figure 5:**
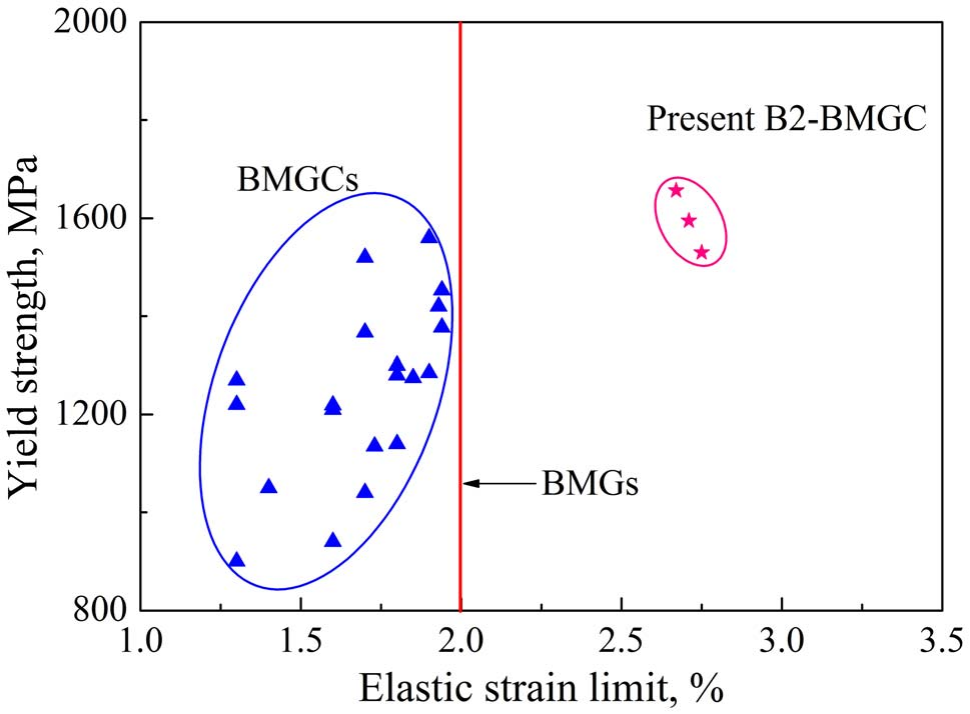
Plot of tensile yield strength-elastic strain limit for previous reported BMGCs^15^,^16^,^17^,^18^,^19^,^22^,^42^,^43^ and the present B2-BMGCs subjected to tensile deformation. It shows that the plastically predeformed B2-BMGC possesses a large elastic strain limit and a relatively high strength.

Figure 5 shows the tensile yield strength-elastic strain limit data from previous reported Cu-, Zr-, and Ti-based BMGCs and the present B2-BMGC. The line *ε* = 2.0% is a typical elastic strain limit for monolithic BMGs. For the previous reported BMGCs^15^,^16^,^17^,^18^,^19^,^22^,^42^,^43^, they are located on the left of the line *ε* = 2.0%. Their elastic strain limit approximately ranges from 1.4% ~ 1.9%, with yield strength ranging from 900 MPa to 1560 MPa. As to the plastically predeformed B2-BMGC, they are shown on the upper right corner of the diagram, and are obviously away from the line *ε* = 2.0%, exhibiting a good combination of large elastic strain limit and high strength, as shown in Fig. 5a.

In summary, this study demonstrates that the metastable B2 phase can effectively promote multiple shear bands and thus significantly improve the plastic deformation capability of B2-BMGCs, and plastically predeformed B2-BMGCs can exhibit a large elastic strain limit. These unique mechanical properties are attributed to the reversible B2↔B19’ phase transformation and the complicated stress states of the MG matrix and the second phase. This finding implies that the elastic properties of BMGCs can be tailored by carefully choosing the reinforcer, with suitable treatment, and can be potentially used as elastic devices or special elastic structure components in engineering fields.

## Methods

### MGC alloy production

The B2-BMGCs with nominal chemical compositions of Zr_48_Cu_47.5_Al_4_Nb_0.5_ were prepared by arc melting the elements with purity better than 99.9%, and by casting into a copper mold. Ingots of diameter 3 mm and length 85 mm were produced.

### Microstructure characterization

The phases of the BMGC ingots were characterized by X-ray diffraction (XRD) using a Rigaku diffractometer (SmartLab) with Cu *K_α_* radiation and an in-situ loading unit. The structure of the B2/MG interface was observed under a JEM-2100F high-resolution transmission electron microscope (HRTEM). The microstructure was also examined by using a JEM 6490 scanning electronic microscope (SEM) and a Carl Zeiss optical microscope (OM). The volume fraction was determined from the OM images.

### Tensile test

The tensile samples are in a dog-bone shape. Its guage length is 10 mm and the dimension of the cross-section is 1 × 1 mm^2^. The tensile samples were prepared by the electric spark method. The lateral surfaces of all tensile samples were ground and finely polished using a 1.0 μm diamond paste. Tensile tests were conducted in an Instron testing machine at room temperature, using a constant strain rate of 1 × 10^−4^ s^−1^. In determining the tensile properties of the composite, five tensile samples were tested. Their average values and standard deviations were calculated. The deformed samples were investigated by SEM to reveal the deformation and fracture features.

### Finite element modeling

Finite element modeling was utilized to undertake stress analysis for the plastic predeformation of the B2-BMGC. The constitutive equations were directly acquired from the true stress-strain curves of the B2 and MG matrix. The shear stress, von Mises stress and elastic strain of the B2 and MG matrix were measured and compared.

## Author Contributions

F.W. and K.C. designed the study. F.W., S.J., and S.C. conducted the experiments. F.W., K.C., and G.W. analysed the results and wrote the manuscript.
